# Effects of Long-Term Acupuncture Treatment on Resting-State Brain Activity in Migraine Patients: A Randomized Controlled Trial on Active Acupoints and Inactive Acupoints

**DOI:** 10.1371/journal.pone.0099538

**Published:** 2014-06-10

**Authors:** Ling Zhao, Jixin Liu, Fuwen Zhang, Xilin Dong, Yulin Peng, Wei Qin, Fumei Wu, Ying Li, Kai Yuan, Karen M. von Deneen, Qiyong Gong, Zili Tang, Fanrong Liang

**Affiliations:** 1 Acupuncture and Tuina School, Chengdu University of Traditional Chinese Medicine, Chengdu, Sichuan, China; 2 School of Life Science and Technology, Xidian University, Xi'an, Shaanxi, China; 3 School of Clinical Medicine, Chengdu University of Traditional Chinese Medicine, Chengdu, Sichuan, China; 4 Department of Radiology, The Center for Medical Imaging, Huaxi MR Research Center, West China Hospital of Sichuan University, Chengdu, Sichuan, China; 5 German Cancer Consortium (DKTK), Heidelberg, Germany; University of Sevilla, Spain

## Abstract

**Background:**

Acupuncture has been commonly used for preventing migraine attacks and relieving pain during a migraine, although there is limited knowledge on the physiological mechanism behind this method. The objectives of this study were to compare the differences in brain activities evoked by active acupoints and inactive acupoints and to investigate the possible correlation between clinical variables and brain responses.

**Methods and Results:**

A randomized controlled trial and resting-state functional magnetic resonance imaging (fMRI) were conducted. A total of eighty migraineurs without aura were enrolled to receive either active acupoint acupuncture or inactive acupoint acupuncture treatment for 8 weeks, and twenty patients in each group were randomly selected for the fMRI scan at the end of baseline and at the end of treatment. The neuroimaging data indicated that long-term active acupoint therapy elicited a more extensive and remarkable cerebral response compared with acupuncture at inactive acupoints. Most of the regions were involved in the pain matrix, lateral pain system, medial pain system, default mode network, and cognitive components of pain processing. Correlation analysis showed that the decrease in the visual analogue scale (VAS) was significantly related to the increased average Regional homogeneity (ReHo) values in the anterior cingulate cortex in the two groups. Moreover, the decrease in the VAS was associated with increased average ReHo values in the insula which could be detected in the active acupoint group.

**Conclusions:**

Long-term active acupoint therapy and inactive acupoint therapy have different brain activities. We postulate that acupuncture at the active acupoint might have the potential effect of regulating some disease-affected key regions and the pain circuitry for migraine, and promote establishing psychophysical pain homeostasis.

**Trial Registration:**

Chinese Clinical Trial Registry ChiCTR-TRC-13003635

## Introduction

Migraine is a common neurological disorder that typically manifests as repeated episodes of moderate or severe unilateral, pulsating headache aggravated by routine physical activity and is associated with nausea and/or phonophobia and photophobia [1]. Migraine has attracted more and more attention worldwide as a public health issue because of its high prevalence, frequent attack history, significant medical burden, and a serious reduction in quality of life (QOL) and productivity [2], [3]. Although the exact mechanism of migraine is still unclear, there is plenty of neuroimaging evidence showing that migraine is a central nervous system disorder [4]–[6]. Our research group involving migraine without aura patients showed that abnormal structure and function was possibly associated with an impaired pain processing and modulatory process, such as in the anterior cingulate cortex (ACC), insula, basal ganglia, thalamus, supplementary motor area (SMA), prefrontal cortex, etc. [7]–[9].

Acupuncture has a long history in China as one of the treatment modalities of Traditional Chinese Medicine (TCM) and is increasingly being adopted in the West as a complementary and alternative treatment to prevent migraine attacks and to relieve pain during a migraine. The latest Cochrane meta-analysis suggests that acupuncture as a migraine prophylaxis is safe and at least as effective, if not more effective, than prophylactic drug treatment [10]. During the past decade, a considerable number of high quality clinical studies have indicated that acupuncture is able to alleviate headache degree and/or improve the QOL [11]–[13]. However, despite the popularity of acupuncture in migraine therapy, there persists limited knowledge on the physiological mechanisms behind this method, and some controversy on the superiority of verum acupuncture over sham control. Some studies suggested that the obvious influence of acupuncture on pain symptoms was either insignificant or a placebo effect [12], [14].

With the development of neuroimaging techniques, this has provided a brand new view to explore the central mechanisms of acupuncture, and has been a global trend in acupuncture research. We detected cerebral glucose metabolism after short periods of acupuncture stimulation in migraineurs through positron emission tomography (PET) with computed tomography examination, and found that transientapp:addword:transient acupuncture stimulation induced different levels of cerebral glucose metabolism in some pain-related brain regions [15]. In fact, one session of acupuncture stimulation did not fully model the clinical situation, and was hardly enough to achieve the expected effect in clinical practice. Therefore, the cumulative therapeutic effect of long-term acupuncture would help to reveal the underlying mechanisms of acupuncture treatment in more depth.

In the current study, we performed a ReHo approach [16] to compare the blood oxygen level-dependent (BOLD) signals in the brains of migraine patients during the resting-state. ReHo is based on a data-driven approach and thus requires no prior knowledge and has good test-retest reliability [17]. It was originally proposed for measuring the degree of regional synchronization of functional magnetic resonance imaging (fMRI) time courses and focused on the similarities or coherence of the intraregional spontaneous low-frequency (<0.08 Hz) BOLD signal, which enables a novel perspective to understand the functional regulation in particular brain regions. An important advantage of using the ReHo method over other methods is that it detects changes or modulations that are induced by different conditions across the whole brain in a voxel-by-voxel manner. ReHo analysis has been used to study migraine in our group [7], [18], and other diseases like Alzheimer's disease [19], Parkinson's disease [20], attention-deficit/hyperactivity disorder [21], and so on.

We hypothesized that if acupuncture therapy is effective, it would modulate disease-affected brain regions and dysfunctional pain modulatory circuitry in migraine patients. In the current study, a randomized controlled trial and resting-state fMRI were adopted to compare the difference in brain activation patterns evoked by active acupoints and inactive acupoints for migraine patients. Furthermore, a correlation analysis was performed to investigate the possible correlation between clinical variables and brain activity.

## Methods

The protocol for this trial and supporting CONSORT checklist are available as supporting information; see Checklist S1 and Protocol S1. This trial was performed at the Teaching hospital of Chengdu University of TCM. The study protocol was registered with the Chinese Clinical Trial Registry (ChiCTR) (Identifier: ChiCTR-TRC-13003635). The study was performed according to the principles of the Declaration of Helsinki (Edinburgh version, 2000), and was approved by the ethics committee at the Teaching Hospital of Chengdu University of TCM. Based on the previous report about minimum sample size in neuroimaging studies [22], a sample size of 16 per group was needed (total N = 32). Considering a conservative dropout rate of 25%, a total sample size of 40 migraineurs was determined. However, during the period of recruitment, a large number of eligible migraine patients (far more than the originally planned sample size) were willing to receive acupuncture treatment. According to a previous study [11], the difference in mean score of VAS between the acupuncture group and sham acupuncture group at 8 weeks was 1 (δ = 1). For this study, it was determined prospectively that α = 0.05(two-sided), 1-β = 0.9, and that the standard deviation would be 1.2 according to the two group subsets. Thirty-one participants were required for each group (1∶1 allocation). Thus, we decided to enroll a total of 80 participants (after attrition) and randomly selected 40 migraineurs to implement the fMRI experiment.

### 2.1 Participants

All subjects gave written, informed consent after the experimental procedures had been fully explained. Subjects were enrolled from the neurology department of the Teaching Hospital of Chengdu University of TCM. Recruitment took place June 2012 through March 2013. The diagnosis of migraine without aura was established according to the classification criteria of the International Headache Society (IHS) [1]. The inclusion criteria were as follows: (1) all subjects were right-handed and had 2 to 6 migraine attacks per month during the last 3 months and during the baseline period (4 weeks before enrollment); (2) all subjects were 18 to 55 years of age; in addition, the start of headache needed to be before the age of 50; (3) received education for more than 6 years and completed the baseline headache diary; (4) had not taken any prophylactic headache medicine or any acupuncture treatment during the last 3 months; (5) no record of long-term analgesics consumption; and (6) had no contraindications to exposure to a high magnetic field. General exclusion criteria were: (1) existence of neurological diseases; (2) hypertension, diabetes mellitus, hypercholesteremia, vascular/heart disease, and major systemic conditions; (3) pregnant or lactating women; (4) alcohol or drug abuse; (5) any neuroimaging research study participation during the last 6 months; and (6) inability to understand the doctor's instructions.

### 2.2 Study Design

We performed a single-blind, randomized controlled trial with two groups: active acupuncture group and inactive acupuncture group. The primary objective of this study was to compare the difference in resting-state brain activation patterns evoked by active acupoints and inactive acupoints in migraine patients via fMRI assessment. The secondary objective was to investigate the possible correlation between brain responses and clinical efficacy. The total observation period within this study was 12 weeks for each patient, including a baseline period of 4 weeks, and a treatment period of 8 weeks. Headache diaries were given to recruited patients to record the details of migraine attacks for 4 weeks (−4 to 0 weeks) during the baseline period. After the initial assessment and screening, patients who met the inclusion criteria were randomly assigned into the active acupoint group or the inactive acupoint group in a 1∶1 ratio. All patients were asked to document their headache diaries, and the outcome measurement was completed both for the baseline, 4 and 8 weeks after randomization. Additionally, 20 migraineurs in each group were randomly selected to receive fMRI examinations at the end of baseline and at the end of the treatment period respectively.

### 2.3 Randomization

Randomization numbers of 80 patients were generated through computerized block-randomization with the SAS procedure PROC PLAN in the SAS package (SAS Version 9.0, SAS Institute, Inc., Cary, NC) by an independent statistician. In this study, the block size was set to 4, and the number of blocks was 20. Opaque, sealed envelopes with consecutive numbers were used for allocation concealment. Investigators who selected the eligible participants after baseline screening opened the envelopes according to the patients' screening sequence numbers, and placed the patients into either the active group or the inactive group. Additionally, we used Microsoft Excel's sampling tool to generate a random sample of 20 from 40 eligible migraineurs for each group. In the new random list, each number represented the enrolled sequence number in the subgroup. Next, the corresponding patients were selected to perform the fMRI scans.

### 2.4 Intervention

**Figure 1:**
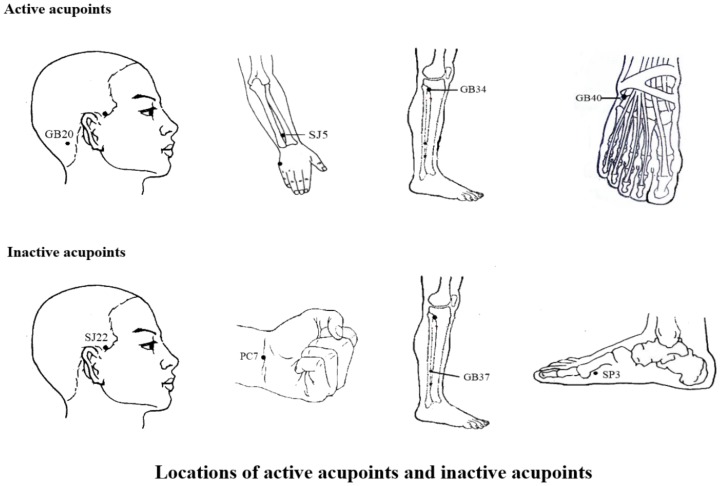
Locations of active acupoints and inactive acupoints. The active acupoints were located as follows: SJ5, on the dorsal aspect of the forearm on the line connecting SJ4 and the tip of the elbow, 2 cun above the transverse crease of the wrist between the ulna and radius; GB20, in a depression between the upper portion of the sternocleidomastoid muscle and the trapezius; GB34, on the lateral aspect of the lower leg in the depression anterior and inferior to the head of the fibula; GB40, anterior and inferior to the external malleolus in a depression on the lateral side of the tendon of the extensor digitorum longus. The inactive acupoints were located as follows: SJ22, on the side of the head on the posterior border of the hairline of the temple at the level with the root of the auricle, posterior to the superficial temporal artery; PC7, in the middle of the transverse crease of the wrist between the tendons of the palmaris longus and flexor carpi radialis; GB37, on the lateral aspect of the lower leg 5 cun above the tip of the external malleolus on the anterior border of the fibula; SP3, proximal and inferior to the head of the 1st metatarsal-phalangeal joint in a depression at the junction of the red and white skin.

In this study, traditional Chinese style acupuncture was used and treatments were manipulated by two specialized acupuncturists with at least five years of training and three years of experience. They received special training prior to the study to ensure they had consistent manual acupuncture therapy. The training program included some standard operation procedures on the locations of the acupoints, acupuncture manipulation techniques, and so on. They implemented acupuncture therapy in both groups by turns. The active acupuncture points were selected according to traditional classic and systematic reviews of ancient and modern literature of acupuncture for migraine upon several consensus meetings with experts based on the experience from our previous study [11], [23]. Moreover, the control group was given inactive acupoints which were chosen according to their anatomical locations, corresponding to Chinese meridians, proximity to verum acupoints and role in treating diseases [24]. The active treatment (group A) was performed on bilateral SJ5 (Waiguan), GB20 (Fengchi), GB34 (Yanglingquan), and GB40 (Qiuxu); and the inactive control (group B) was implemented on bilateral SJ22 (Erheliao), PC7 (Daling), GB37 (Guangming), and SP3 (Taibai) (figure 1).

All acupoints were punctured bilaterally using single-use stainless steel filiform needles (Hwato Needles, Sino-foreign Joint Venture Suzhou Hua Tuo Medical Instruments Co., China), 25 mm–40 mm in length and 0.25 mm–0.30 mm in diameter. The depths of the inserted needles differed but were approximately 2.5 cm–3.5 cm. Needles were twisted with rotation (90°<amplitude<180°) at a frequency of 1–2 Hz. Stimulation was repeated 1–3 times to acquire the *de-qi* sensation (“*de-qi* sensation” is a complex feeling including soreness, numbness, heaviness, distention and dull pain at the site of needle placement). Each group's treatment consisted of 32 sessions of acupuncture over a period of 8 weeks (once every other day, preferably 4 times a week), and each session lasted 30 minutes.

### 2.5 Blinding

Due to the procedure of the acupuncture technique, two acupuncturists in this study were not blinded. Investigators in charge of patient screening and randomized distribution were not involved in treatment and data analyses. They knew the group assignment, but they did not know the corresponding treatment schedule. The outcome assessor, who was not involved in acupuncture treatment and data analyses, was blinded throughout the study.

To guarantee that the patients were blinded during the treatment period, several approaches were performed for migraine patients in both groups: they were informed that they would receive one of two types of acupuncture treatment, which depended on different traditional Chinese acupuncture theories; acupuncture treatment was achieved in a large independent single-room with screen dividers for patient blinding and privacy; and two groups of patients received bilateral and equivalent number of acupoint stimulations each time.

### 2.6 Outcome Measures in Clinical Efficacy

All patients were required to fill out headache diary records for 12 weeks, including a 4-week baseline, and 4 and 8 weeks after randomization. The headache diary recorded the severity, frequency and duration of headache according to the guidelines of the IHS for Clinical Trials in Migraine [25]. VAS score 0–10 as a primary clinical outcome measured the intensity of headache. As secondary clinical outcome measures, the number of days with a migraine per 4 weeks and frequency of migraines per 4 weeks (defined as the number of migraine separated by pain free intervals of at least 48 hours) measured the duration and severity of headache respectively. In addition, the HIT-6 questionnaire [26] was adopted to assess the severity and impact of headache on a patient's life.

It is worth mentioning that the feelings of *de-qi* were collected after removing needles during the 8^th^, 16^th^, 24^th^, and 32^nd^ sessions. Migraineurs were interviewed by an acupuncturist who did not know the treatment allocation. Patients were asked to evaluate each component of the *de-qi* sensations they had experienced during the acupuncture stimulation period, and the intensity used a VAS ranging from 0 (none) to 10 (max), which has been commonly used to measure the feelings of *de-qi* [27], [28]. The score for the VAS was the sum of all component scores. The overall *de-qi* score was the mean score from all sessions.

### 2.7 fMRI data acquisition

Resting-state fMRI scans were performed on each group at the baseline and after 8 weeks' treatment to detect the local features of spontaneous brain activity. The imaging data were carried out in a 3 Tesla Siemens MRI system (Allegra, Siemens Medical System, Erlangen, Germany) at the Huaxi MR Research Center, West China Hospital of Sichuan University, Chengdu, China. A standard eight-channel phase-array head coil was used, along with restraining foam pads to minimize head motion and to diminish scanner noise. The resting-state functional images were obtained with echo-planar imaging (EPI) (30 continuous slices with a slice thickness = 5 mm, repetition time = 2000 ms, echo time = 30 ms, flip angle = 90°, field of view = 240 mm×240 mm, matrix = 64×64). During the 6-min fMRI scanning, participants were instructed to keep their eyes closed, relax, move as little as possible, and stay awake. It needs to be emphasized that if there was an attack for migraine patients during the scan or examination, they could not be scanned and the scan would be postponed. In this study, records in the headache diary were checked to ensure every patient did not suffer from a migraine attack at least 72 hours prior to the brain scan.

### 2.8 Data Analysis

#### 2.8.1 Clinical data analysis

The statistical analysis was performed by an independent statistician blinded to treatment allocation in the Teaching Hospital of Chengdu University of TCM. SPSS statistical package program (Version 14.0, SPSS Inc., Chicago, IL, USA) was used. Baseline characteristics and clinical outcomes were analyzed by the intention-to-treat (ITT) population which included all participants who had randomized allocation. Missing data of dropped-out participants were replaced by the last observation carried forward (LOCF) method. The significant level used for the statistical analysis with 2-tailed testing was 5%. Continuous variables were presented as the mean (standard deviation) with 95% confidence intervals (CI). Categorical variables were described as n (percentage). Treatment effects such as VAS, frequency of migraine attack per 4 weeks, number of days with migraine per 4 weeks, and HIT-6 were evaluated using a repeated-measures analysis of variance (ANOVA) model with a between-subjects factor Therapy (levels: active and inactive) and a within-subjects repeated measures factor TIME (levels: baseline, 1–4 weeks, and 5–8 weeks). For the change in VAS, analysis of covariance with baseline VAS as a covariate was used to compare the difference between two groups at the end of treatment. The general linear model repeated measures procedure was used to test the differences in the repeated continuous variables (*de-qi* sensations) between the two groups.

#### 2.8.2 Imaging data preprocessing

In the functional image data preprocessing, the first five scans were discarded to eliminate nonequilibrium effects of magnetization and to allow participants to become familiar with the scanning circumstances. Data preprocessing was done using Statistical Parametric Mapping (SPM5, http://www.fil.ion.ucl.ac.uk/spm). The images were corrected for the acquisition delay between slices, aligned to the first image of each session for motion correction and spatially normalized to the standard Montreal Neurological Institute (MNI) template in SPM5. We calculated the maximum excursion movement values for each of the translation planes (x, y, and z) and each of the rotation planes (roll, pitch, and yaw) for every participant. None of them had head movements exceeding 1 mm on any axis and head rotation greater than 1° during the entire fMRI scan. Finally, a band-pass filter (0.01 Hz<f<0.08 Hz) was applied to remove physiological and high-frequency noise.

#### 2.8.3 MRI data analysis

ReHo, a method proposed by Zang et al. [29], was performed in the Resting-state fMRI Data Analysis Toolkit (http://www.restfmri.net) [16]. First, the Kendall's coefficient of concordance (KCC) of each voxel was calculated by the time series of the voxel and its nearest 26 neighboring voxels (cluster size = 27). Second, the KCC maps were standardized by their own mean KCC within the whole brain mask. Third, the resulting maps were smoothed with a Gaussian kernel with a full-width at half-maximum (FWHM) of 4 mm. In the statistical analysis, an independent-sample *t*-test was used to explore ReHo differences between the two groups with age as a covariate. Results were assumed to be statistically significant at *P*<0.05 after false discovery rate (FDR) correction within the whole brain. The correlation analysis was performed based on different clusters in the brain after acupuncture treatments relative to the baseline for each group. Within each cluster, we extracted the ReHo values after acupuncture and baseline respectively. The mean of their subtraction (end of treatment-baseline) was correlated with the changes in the clinical variables, and Bonferroni correction was used.

## Results

### 3.1 Participants

**Figure 2:**
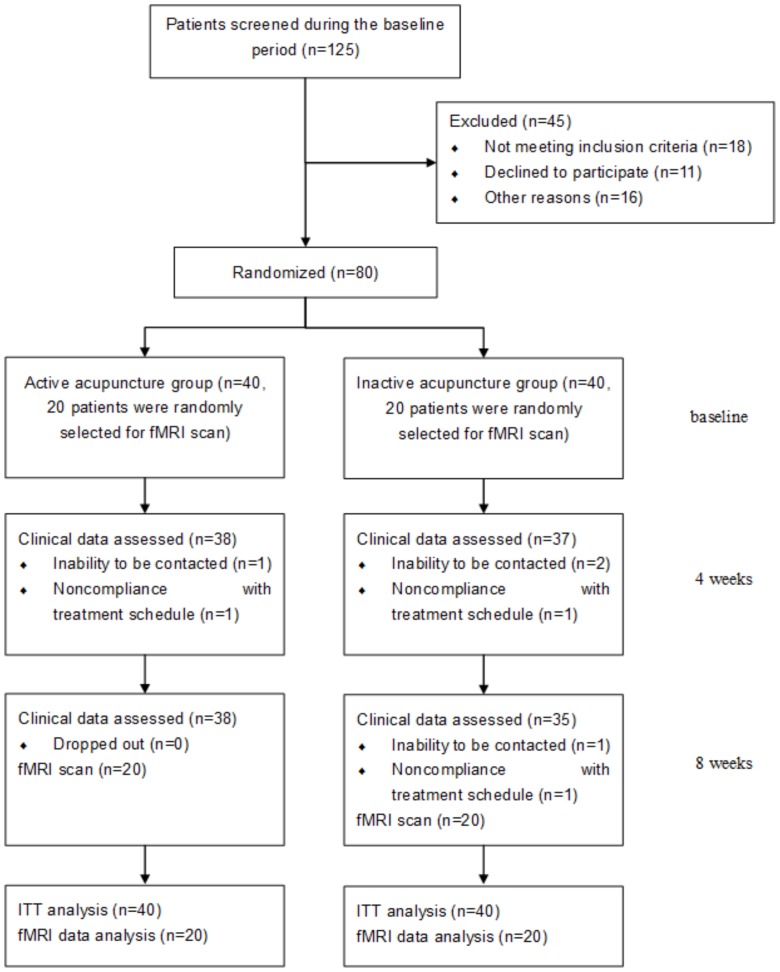
The flow chart of study. The flow chart of this study according to the CONSORT Statement.

Eighty eligible patients were equally allocated into the active treatment group and inactive treatment group (40 in each group). Two patients from the active acupuncture group and five from the inactive acupuncture group dropped out during the study because of private reasons: noncompliance with treatment schedule or inability to be contacted (figure 2). In total, 80 patients who received acupuncture therapies were included in the ITT analysis of the clinical outcome measures. The baseline and demographics with the ITT population are shown in table 1, which showed that the two groups were comparable at baseline. Furthermore, 40 patients (20 in each group) finished the fMRI scans, and the baseline characteristics did not differ between the two groups (table 2).

**Table 1:** Baseline and demographics for migraine patients without aura (ITT).

Items	Active acupoint Group (n = 40)	Inactive acupoint Group (n = 40)
Mean age (SD), (years)	33.35 (11.69)	33.23 (9.73)
Female, n (%)	28 (70.0)	29 (72.5)
Mean education(SD), (years)	12.70 (3.29)	13.68 (3.74)
Mean duration of illness (SD), (years)	10.58 (7.40)	9.93 (5.73)
Family history (Y (%)/N (%))	8 (20.0)/32 (80.0)	9 (22.5)/31 (77.5)

Notes: ITT, intention-to-treat; SD, Standard deviation; Y, yes; N, no.

**Table 2:** Baseline characteristics of 40 migraineurs who participated in the fMRI scan.

Items	Active acupoint Group (n = 20)	Inactive acupoint Group (n = 20)
Mean age (SD), (years)	32.90 (10.99)	37.25 (9.68)
Female, n (%)	14 (70.0)	12 (60.0)
Mean education(SD), (years)	12.95 (3.52)	13.35 (4.12)
Mean duration of illness (SD), (years)	8.55 (6.49)	10.40 (7.40)
Family history (Y (%)/N (%))	2(10.0)/18 (90.0)	0 (0)/20 (100.0)
VAS score (SD)	5.28 (2.03)	5.44 (1.48)
Frequency of migraine attacks per 4 weeks*	7.90 (4.88)	5.45 (4.33)
Number of days with migraine (days) per 4 weeks	11.45 (9.30)	8.75 (9.21)
HIT-6 score	60.45 (8.13)	61.55 (7.98)

Notes: SD, Standard deviation; Y, yes; N, no.;

*Frequency of migraine attack, the number of episodes of migraine attacks separated by pain-free intervals of at least 48 hours.

### 3.2 Neuroimaging results

**Figure 3:**
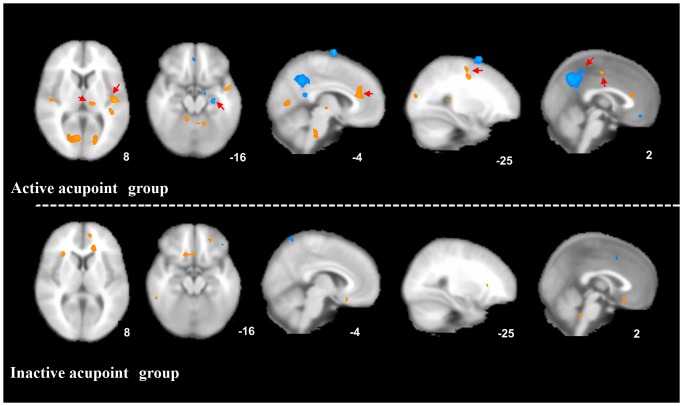
Brain activity in migraineurs without aura after different acupuncture treatment. Long-term active acupoint therapy elicited a more extensive and remarkable cerebral response compared with acupuncture at inactive acupoints.

In the active acupoint group, migraine patients showed significantly higher ReHo values in the bilateral ACC (Brodmman area (BA) 24, BA32), insula (BA13), thalamus, SMA (BA6), superior temporal gyrus (STG) (BA22), cuneus (BA17, BA18), lingual gyrus (BA18), cerebellum, and brainstem after acupuncture treatment. A decrease in ReHo values was observed after treatment in the bilateral posterior cingulate cortex (PCC) (BA31), middle frontal gyrus (MFG) (BA10), angular gyrus (BA39), precuneus (BA7), middle temporal gyrus (MTG) (BA39), left hippocampus, inferior parietal lobule (BA39), inferior temporal gyrus (ITG) (BA20), and right postcentral gyrus (BA40) (*P*<0.05, FDR corrected with a minimal cluster size of 20 voxels) (table 3 and figure 3).

**Table 3:** The cerebral ReHo changes in migraine patients without aura after active or inactive acupuncture treatment.

Region	Hemi	Active acupoint Group (n = 20)							Inactive acupoint Group (n = 20)						
		Talairach			*t* value	BA	Sign	Cluster size	Talairach			*t* value	BA	Sign	Cluster size
		x	y	z					x	y	z				
**Limbic System**															
ACC	L	−3	30	12	3.52	BA24/32	**↑**	33	−6	46	−5	3.47	BA32	**↑**	23
	R	6	33	23	3.86		**↑**	52							
PCC	L	−3	−45	41	−3.78	BA31	**↓**	39							
	R	3	−48	38	−3.78		**↓**	61							
Hippocampus	L	−30	−18	−14	−3.84	-	**↓**	21							
	R														
Insula	L	−42	−11	6	3.26	BA13	**↑**	21							
	R	36	−14	20	4.04		**↑**	23							
Thalamus	L	−15	−20	4	3.95	VPM	**↑**	26							
	R	15	−20	7	2.89		**↑**	21							
	L	−18	−20	4	3.05	VPL	**↑**	22							
	R	15	−17	4	3.34		**↑**	27							
**Frontal Cortex**															
MFG	L	−21	62	22	−3.37	BA 10	**↓**	40							
	R	42	59	14	−4.47		**↓**	79	45	2	44	−4.43	BA6	**↓**	32
MeFG	L								−6	49	−5	3.69	BA10	**↑**	26
	R														
SMA	L	−27	−9	50	2.9	BA6	**↑**	26							
	R	33	−9	53	3.78		**↑**	47							
**Temporal Cortex**															
STG	L	−45	−18	−2	4.76	BA 22	**↑**	67							
	R	50	−9	0	5.38		**↑**	87							
MTG	L	−50	−63	28	3.56	BA 39	**↓**	30							
	R	50	−63	28	4.2		**↓**	53							
ITG	L	−56	−10	−27	−3.29	BA 20	**↓**								
	R														
**Occipital Cortex**															
Cuneus	L	−18	−78	9	3.71	BA 17/18	**↑**	51							
	R	21	−86	21	3.94		**↑**	85							
Lingual gyrus	L	−15	−73	4	2.84	BA 18	**↑**	16							
	R	12	−73	4	3.55		**↑**	71							
**Parietal Lobe**															
Inferior parietal lobule	L	−45	−65	42	−3.94	BA39	**↓**	37							
	R														
Angular gyrus	L	−48	−71	31	−4.21	BA39	**↓**	39							
	R	50	−65	31	−3.67		**↓**	31							
Postcentral gyrus	L														
	R	56	−33	49	−3.11	BA 40	**↓**	28							
Precuneus	L	−3	−44	46	−3.72	BA7	**↓**	153							
	R	3	−54	36	−4.58		**↓**	47							
**Cerebellum**	L	−15	−53	−12	3.89	-	**↑**	73							
	R	12	−47	−13	3.61		**↑**	37							
**Brainstem**	L					-									
	R	6	−34	−31	3.96		**↑**	51							

Notes: *P*<0.05, FDR corrected with a minimal cluster size of 20 voxels; Hemi, Hemisphere; BA, Brodmann Area; Up or down arrow (↑/↓) indicates whether the structure showed a signal increase or decrease respectively; L, left; R, right.

In the control group, an increase in ReHo values was observed after inactive treatment in the left ACC (BA32) and medial frontal gyrus (MeFG) (BA10). A signal decrease in ReHo values was detected in the right MFG (BA6) (*P*<0.05, FDR corrected with a minimal cluster size of 20 voxels) (table 3 and figure 3).

**Figure 4:**
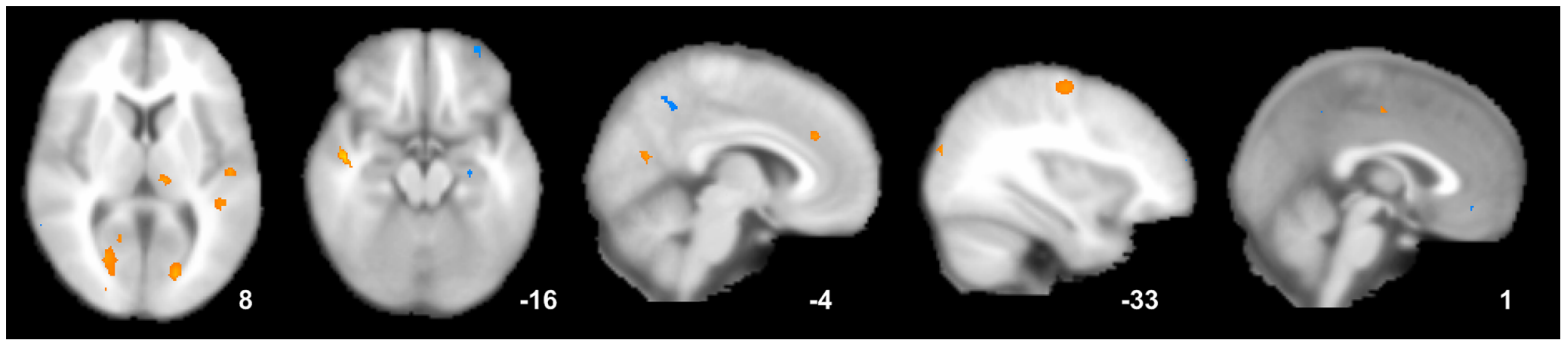
Direct comparison of the ReHo changes between the active and inactive group. The active acupoint group showed higher ReHo in the thalamus, ACC, superior temporal gyrus, SMA and lower ReHo in the hippocampus, middle frontal gyrus, and middle temporal cortex than the inactive group (*P*<0.001, uncorrected).

Additionally, we have performed a direct comparison of the ReHo changes between the active and inactive group. The active acupoint group showed higher ReHo in the thalamus, ACC, STG, SMA and lower ReHo in the hippocampus, MFG, and MTG than the inactive group (*P*<0.001, uncorrected) (as shown in figure 4).

### 3.3 Clinical outcomes and comparison of *de-qi* sensations

Comparison within each group, both the active acupoint group and inactive group showed significant decreases in the VAS score, frequency of migraine attack per 4 weeks, number of days with migraine per 4 weeks and HIT-6 score after 8 weeks' treatment (*P*<0.05). Based on this study, a significant difference was found in the VAS scores between the two groups by analysis of variance for repeated measures (*P* = 0.015) (table 4). The difference in VAS between the active group and inactive group was more than 0.9 in week 8 (*P* = 0.006). However, no significant differences were observed between the two groups for the frequency of migraine attack per 4 weeks, number of days with migraine per 4 weeks, and HIT-6 score at the end of treatment (*P*>0.05) (table 4). Furthermore, analysis of variance of repeated measures indicated that there was no significant difference between the two groups in *de-qi* sensations (*P*>0.05) (table 5).

**Table 4:** Clinical outcome measures in each group (ITT).

	Active acupoint Group (n = 40)		Inactive acupoint Group (n = 40)			
Outcome measure	Mean (SD)	95% CI	Mean (SD)	95% CI	*P* ^¶^	*P* ^†^
VAS score						
−4–0 weeks	5.11 (1.75)	(4.55–5.67)	5.23 (1.78)	(4.66–5.80)	0.7484	*P*_T_ = 0.0000
1–4 weeks	3.80 (1.62)	(3.28–4.32)	4.64 (1.17)	(4.27–5.01)	0.0094	*P*_T_ *_G_ = 0.0888
5–8 weeks	3.07 (1.57)	(2.57–3.57)	4.07 (1.54)	(3.58–4.56)	0.0052	*P_G_* = 0.0150
Difference from baseline in VAS∥	2.096 (0.25)	(1.61–2.58)	1.110 (0.25)	(0.62–1.60)	0.006‖	-
Frequency of migraine attacks per 4 weeks*						
−4–0 weeks	6.83 (4.21)	(5.48–8.17)	5.98 (3.72)	(4.79–7.16)	0.3412	*P*_T_ = 0.0000
1–4 weeks	4.35 (2.63)	(3.51–5.19)	3.92 (1.69)	(3.38–4.46)	0.3802	*P*_T_ *_G_ = 0.3168
5–8 weeks	2.85 (2.19)	(2.15–3.55)	3.10±2.00	(2.46–3.74)	0.5983	*P_G_* = 0.4742
Number of days with migraine (days) per 4 weeks						
−4–0 weeks	9.85 (7.94)	(7.31–12.39)	9.73 (7.62)	(7.29–12.16)	0.9429	*P*_T_ = 0.0000
1–4 weeks	5.56 (4.25)	(4.20–6.92)	4.91 (2.36)	(4.16–5.66)	0.4043	*P*_T_ *_G_ = 0.6459
5–8 weeks	3.51 (2.66)	(2.66–4.36)	3.91 (2.82)	(3.01–4.82)	0.5122	*P_G_* = 0.8835
HIT-6 score						
−4–0 weeks	58.10 (6.81)	(55.92–60.28)	58.13 (7.12)	(55.85–60.40)	0.4224	*P*_T_ = 0.0000
1–4 weeks	47.25 (9.55)	(44.20–50.30)	49.69 (9.35)	(46.70–52.68)	0.2515	*P*_T_ *_G_ = 0.3834
5–8 weeks	47.86 (8.42)	(45.17–50.55)	50.39 (6.67)	(48.26–52.52)	0.1395	*P_G_* = 0.2232

Notes: ITT, intention-to-treat;

CI, confidence interval;

*Frequency of migraine attack, the number of episodes of migraine attacks separated by pain-free intervals of at least 48 hours;

¶ *P* values based on *t*-test between the two groups;

† *P* values based on repeated measures;

∥ based on analysis of covariance analysis;

*P*_T_, values for comparison between different time points;

*P*_T*G_, values for Time*Group interaction;

*P_G_*, values for comparison between different groups.

**Table 5:** Comparison of *de-qi* sensations during treatment period (ITT).

Time points	Active acupoint Group (n = 40)	Inactive acupoint Group (n = 40)	*P* value
1^st^	8.70±4.16	10.31±4.26	0.1070
2^nd^	8.61±4.63	8.09±4.64	0.6372
3^rd^	9.13±3.89	8.18±3.70	0.2890
4^th^	10.62±4.45	10.42±4.42	0.8461

Notes: Comparison between different time points: F = 4.128, *P* = 0.007; Time*Group: F = 1.384, *P* = 0.249; Comparison between different groups: F = 0.001, *P* = 0.9790.

### 3.4 Correlation coefficients of the brain response and clinical variable results

**Figure 5:**
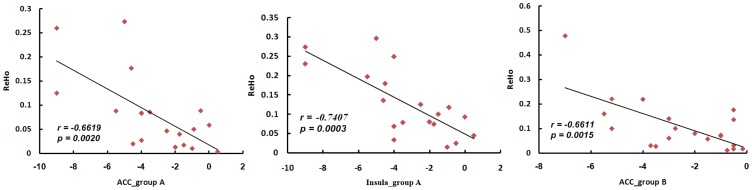
Correlation coefficients of brain response and VAS score. A. Active acupoint group; B. Inactive acupoint group. The decrease in the VAS score was significantly related to the increased average ReHo values in the ACC in the two groups (*P*<0.05, Bonferroni corrected). Moreover, the decrease in the VAS score was associated with increased average ReHo values in the insula (*P*<0.05, Bonferroni corrected) which could be detected in the active acupoint group.

In the active acupoint group, the decrease in the VAS score was significantly related to the increased average ReHo values in the ACC (r = −0.6619, *P*<0.05) and insula (r = −0.7407, *P*<0.05, Bonferroni corrected). In the inactive control group, the decrease in the VAS score was only significantly related to the increased average ReHo values in the ACC (r = −0.6611, *P*<0.05, Bonferroni corrected) (figure 5).

### 3.5 Adverse events

No serious adverse events happened during the study. One case in the active acupuncture group suffered acupuncture fainting during acupuncture treatment. The patient was told to lie down and rest. The symptoms of dizziness and sweating disappeared in 15 minutes. Two cases in each group reported having minor hemorrhage at the needling site. They were told to put pressure on the needling areas for about 5 minutes, and recovered in a short time. All of the patients with adverse events completed the study process.

## Discussion

In this RCT study, we focused on the difference in brain activation patterns evoked by active acupoints and inactive acupoints in migraine patients via fMRI assessment, and determined the potential physiological mechanism behind this therapy. An inactive acupoint is a validated sham control method in acupuncture research [30]–[32] with the advantage of minimizing bias from patients. By the way, non-acupoints are thought to have no therapeutic influence and are usually adopted as a placebo control in previous clinical trials and neuroimaging studies. In addition, minimal acupuncture or superficial insertion was often employed to stimulate non-acupoints producing inconspicuous *de-qi* sensations [11], [23], but this might significantly cause bias among Chinese subjects. To ensure comparability between the two groups during acupuncture manipulation, *de-qi* sensations were assessed several times during the treatment session. Needling at inactive acupoints could effectively reduce the aforementioned bias, and evenly control non-specific factors such as expectancy effects during the period of study. In this experiment, the overall *de-qi* sensations in the active acupoint group and inactive acupoint group were comparable and had no statistical difference.

### 4.1 Similarities and differences in clinical efficacy between active acupuncture and inactive acupuncture

Based on the clinical outcomes of this RCT, both active and inactive acupuncture methods were helpful in treating migraine after 8 weeks of therapy (*P*<0.05). Both treatments remarkably alleviated the clinical symptoms of migraine (intensity of pain, attack frequency, and days with migraine) and improved the QOL. Furthermore, acupuncture at active acupoints was significantly superior to acupuncture at inactive acupoints in alleviating pain intensity (*P* = 0.015) in the current study. This result was similar with our previous RCT report on the efficacy of acupuncture at true acupoints compared with non-acupoints for migraine prevention [11]. We inferred that similar clinical effects of both treatments might partly result from placebo and psychological effects. The placebo response is an essential part of pain treatment, especially in the improvement of headache sufferers. A systematic review has shown that when clinicians stated positive outcome expectancies as opposed to uncertain expectancies, most studies found improvements in patient self-reports on pain, anxiety, and distress [33]. During the process of the study, two acupuncturists were responsible for the treatments alternately, and another experienced doctor who did not know the treatment allocation took charge of the efficacy evaluation. As we know, acupuncture treatment could create enhanced placebo effects, such as patient expectations, longer patient-doctor appointments, and the power of touch and suggestion, so both the active treatment and inactive treatment evenly ameliorated the headache degree and frequency originating from patients' self-reports which may be explained by the aforementioned nonspecific effects.

### 4.2 The similarities in resting-state brain activity evoked by active and inactive treatment

Based on the resting-state fMRI results, common brain regions responding to the acupuncture active treatment and inactive treatment included the ACC, MFG, and MTG. Among these areas, there was a significant negative correlation between the increased average ReHo values of the ACC and a decrease in the VAS score in both groups (*P*<0.05, corrected). The results suggested that the increase in ReHo values in the ACC might be the common mechanism of acupuncture treatment for migraine patients, despite the needled active acupoints or inactive acupoints.

The ACC is a key region composed of the “pain matrix” and is involved in the medial pain system. It is one of the common “brain signature” structures in chronic pain diseases, and is thought to be engaged with both cognitive-attentional and affective dimensions of pain. The ACC has been recognized in playing a deterministic role in endogenous pain control, which is mediated by endogenous opioid systems [34]. In previous neuroimaging studies, the ACC was the most consistently deactivated region in PET and fMRI migraine studies [35], [36], and also had a decrease in gray matter [37], [38]. Our research group verified that compared with healthy controls, migraineurs showed a significant decrease in ReHo values and amplitude of low-frequency fluctuation (ALFF) in the ACC [7], [9], and showed aberrant functional connectivity which had the ACC involved [8], [39]. In the present study, acupuncture-induced reduction in pain intensity ratings was negatively associated with increased average ReHo values in the ACC which illustrated that acupuncture treatment could promote pain reduction successfully by modulating the migraine-affected dysfunction region, the ACC, to some extent.

On the other hand, we inferred that the similarities in both clinical improvements and cerebral responses between active treatment and inactive treatment were possibly due to the placebo effect. During the process of treatment, migraineurs had positive expectations towards acupuncture therapy independent of whether or not the treatments were active or inactive, and moreover, migraineurs were blinded to their groups and acquired comparable *de-qi* sensations throughout the duration of therapy. The ACC was commonly activated by acupuncture stimulation [40], [41], and it plays an important role during placebo modulation of pain perception. Recent papers have described the effect of the placebo and expectancy on acupuncture analgesia, and certain findings were similar to our results. For example, some researchers found that reductions in pain intensity ratings were associated with placebo and opioid analgesia coinciding with increased activity in the ACC [42]. Additionally, active acupoint treatment induced a more prominent cerebral response in the ACC, thus we considered that the differences in both clinical variables and neuroimaging data between the two groups indicated that the placebo effect could not fully explain the neurobiological underpinning of active acupuncture therapy.

### 4.3 The difference in resting-state brain activity evoked by active and inactive treatment

Compared with acupuncture at inactive acupoints, long-term active acupoint therapy elicited a more extensive and remarkable cerebral response in the present study. The following cerebral regions exhibited increases in ReHo values only in the active acupoint group: the bilateral insula, thalamus, SMA, STG, cuneus, lingual gyrus, cerebellum, and brainstem. Meanwhile, decreases in ReHo values in the bilateral PCC, MTG, angular gyrus, precuneus, left hippocampus, ITG, inferior parietal lobule, and right postcentral gyrus were observed only in the active acupoint group (table 3 and figure 3). A further direct between-group comparison indicated that the active acupoint group showed higher ReHo in the thalamus, ACC, SMA, STG and lower ReHo in the hippocampus, MFG, and MTG than the inactive group (figure 4). In general, we found that active acupoint treatment for a longitudinal course had major effects on the pain matrix, lateral pain system, medial pain system, default mode network (DMN), and some regions closely related to the cognitive components of pain processing.

In our results, active acupoint treatment elicited dramatic and extensive ReHo changes in the bilateral thalamus, including the ventral posterolateral nucleus (VPL) and ventral posteromedial nucleus (VPM). These nuclei are key intermediates in the lateral pain system, and play an important role in processing spatial and intensity aspects of noxious stimuli. The thalamus commonly had a dysfunction in migraine patients in previous documents [5], [43], and its abnormalities were deemed to contribute to migraine pathophysiology. Pharmacological studies demonstrated that successful migraine preventive treatments modulated thalamic activity [5]. We speculated that the correlation between the VAS score and ReHo values was not detected in the thalamus, but was attributed to its relay function between a variety of subcortical areas and the cerebral cortex. Our results indicated that active acupoint treatment may play a major role in the sensory-discriminative component of pain and also in the appropriate modulation of the emotional aspect of pain.

In addition, as the core regions of the DMN, the PCC and precuneus were found to have decreased ReHo values after active acupuncture treatment. These findings were consistent with previous evidence which supports the position that genuine acupuncture leads to stronger DMN deactivation than sham acupuncture on healthy subjects [44]. Previous resting-state fMRI studies have shown that various pain diseases were associated with abnormal connectivity patterns among DMN regions [45], [46], and our research group confirmed that migraine patients without aura had a DMN abnormality compared with healthy controls [39]. The DMN has previously been suggested as a potential neural marker of treatment efficacy in chronic pain, and our findings demonstrated that active acupuncture analgesia could be achieved by regulating the migraineurs' resting state and changing the dysfunctional architecture of the DMN.

Based on our results, we detected that a long course of acupuncture treatment on active acupoints affected the hippocampus, which is associated with cognitive components of pain processing, as well as a major component of the human brain that links affective states with memory processing. The hippocampus was described as having increased gray matter volume in patients suffering chronic pain in a meta-analysis [47], and this was confirmed in a recent migraine study [6]. It seems to frequently participate in the central effects of acupuncture. The cerebellum has anatomical connections with multiple areas of the frontal cortex and limbic regions, which are critical for its involvement in emotional and cognitive processing. A previous animal study indicated that the cerebellum contributes more to pain processing than just motor control [48]. Decreased gray matter volume in the cerebellum has been recently described in migraine patients without aura in a voxel-brain morphometry study in our research group [49], and another study also verified the migraineurs' cerebellar microstructural abnormalities [50]. Several independent functional imaging studies have reinforced the fact that the dysfunction of the brain stem is related to the pathogenesis of migraine [51]–[53]. The brain stem serves as a lower center in functions such as pain sensitivity control and consciousness. In our study, the modulation of active acupuncture treatment on the hippocampus, cerebellum and brain stem might be related to regulating the process of nociceptive information and homeostatic emotion originating from pain processing.

### 4.4 The potential mechanism of active acupuncture therapy for migraineurs

In order to better explore the possible physiological mechanism underlying different acupuncture treatments for migraine patients, a correlation analysis was employed. Except for the ACC, which is a co-related brain area for the two groups, we further noted that VAS reduction following active acupoint treatment was associated with the insula. The insula is a functionally heterogeneous brain region that participates in pain perception, emotional processing and interoception. It was commonly revealed that there was a difference in gray matter volume in chronic pain patients compared with healthy controls [47]. The insular networks were found to be altered by migraine headache [54], [55], and our research team demonstrated that migraine patients have dysfunctional connectivity involved with the insula [8], [39]. We were interested in the active acupuncture-induced reduction in pain intensity ratings which were negatively associated with increased average ReHo values in the insula, as well as the ACC in the present study. These two regions belong to the “homeostatic afferent pathway”, which carries information about the physiological status of tissues in the body. Pain is both an aspect of interoception and a behavioral drive caused by a physiological imbalance that homeostatic systems alone cannot rectify [56], [57]. Our results illustrated that active acupuncture treatment could alleviate migraine intensity by modulating the disordered homeostatic afferent network back to physiological balance.

## Limitations

The main limitations of the present study included the following: we expanded the number of eligible participants involved in the RCT, but the fMRI examinations were performed for 40 migraineurs who were randomly selected from 80 eligible migraineurs, so the correlation analysis of clinical measures and brain responses involved only 40 participants who completed the fMRI detections. Lack of an index to access and quantify the expectancy during the acupuncture treatment session is another limitation. Further studies need to quantify the patients' expectation and explore the effect on clinical efficacy and physiological mechanism of some non-specific factors during long-term acupuncture treatment.

## Conclusions

In conclusion, the current study showed that long-term active acupoint therapy and inactive acupoint therapy have different brain activities. Acupuncture at active acupoints might have the potential effect of regulating some disease-affected key regions and the pain circuitry for migraine. More importantly, our results provided some evidence that active acupuncture treatment as a holistic therapy promotes psychophysical pain homeostasis.

## Supporting Information

Checklist S1 **CONSORT Checklist.**
(DOCX)

Protocol S1 **Trial Protocol.**
(DOCX)
