# Supplementary material for: Effects of Long-Term Acupuncture Treatment on Resting-State Brain Activity in Migraine Patients: A Randomized Controlled Trial on Active Acupoints and Inactive Acupoints
Source: PLoS One. 2014 Jun 10;9(6):e99538. doi: 10.1371/journal.pone.0099538 (PMC4051855; doi:10.1371/journal.pone.0099538)
Supplement: Protocol S1 — Trial Protocol. (DOCX) [file pone.0099538.s002.docx]

## The characteristics of central response of acupuncture for treating migraine

## The purpose of study

The purpose of this study is to compare the differences in brain activities evoked by active acupoints, inactive acupoints and acupoints on other meridians to investigate the possible correlation between clinical efficacy and brain responses.

## 2. Object of study

### 2.1 Recruitment

Subjects suffered migraine without aura will be recruited from the neurology department of the Teaching Hospital of Chengdu University of Traditional Chinese Medicine (TCM).

### 2.2 The choice of migraine patients

2.2.1 Diagnostic criteria

The diagnosis of migraine without aura is established according to the classification criteria of the International Headache Society (IHS) published in 2004.

2.2.2 Inclusion criteria

1. match the diagnosis of migraine patients without aura from the classification criteria of the International Headache Society (IHS) aforementioned;
2. all subjects are right-handed, and 2 to 8 migraine attacks per month during the last 3 months and during the baseline period (4 weeks before enrolment);
3. all subjects are 18 to 55 years of age; in addition, start of headache should be before the age of 50 years;
4. have received education for more than 6 years and have completed baseline headache diary;
5. have not taken any prophylactic headache medicine or any acupuncture treatment during the last 3 months;
6. no contraindications for exposure to a high magnetic field;
7. sign the written informed consent.

2.2.3 Exclusion criteria

1. existence of neurological diseases;
2. had hypertension, diabetes mellitus, hypercholesteremia, vascular/heart disease, and major systemic conditions;
3. pregnant or lactating women;
4. alcohol or drug abuse;
5. any neuroimaging research study participation during the last 6 months;
6. inability to understand the doctor’s instructions.

## 3. Method

### 3.1 Instrument and material

Instrument：3 Tesla Siemens MRI system (Allegra, Siemens Medical System, Erlangen, Germany)

Material：single use stainless steel filiform needles (Hwato Needles, Sino-foreign Joint Venture Suzhou Hua Tuo Medical Instruments Co., China); 25 mm-40 mm in length and 0.25 mm-0.30 mm in diameter

### 3.2 Sample size

20 subjects in each group

### 3.3 Study design

Randomization: using a computer-generated randomization sequence

Group assignment:

- Group A: acupuncture at active acupoints
- Group B: acupuncture at inactive acupoints
- Group C:acupuncture at acupoints on other meridians

Patients will be blinded to group assignment. Data management and analysis will be performed by researchers who are blinded to the acupuncture procedures.

Study schedule:

The total observation period within this study is 12 weeks for each patient. All patients will be asked to document their headache diaries, and the outcome measurement is completed both in the baseline and 4 and 8 weeks after randomization. Resting-state fMRI scans will be performed on each group before and after treatment to detect the local features of spontaneous brain activity.

### 3.4 Treatment schedule

3.4.1 Group assignment and acupoint selection

- Group A:SJ5 (Waiguan), GB20 (Fengchi), GB34 (Yanglingquan), GB40 (Qiuxu)
- Group B: SJ22 (Erheliao), PC7 (Daling), GB37 (Guangming), SP3 (Taibai)
- Group C: PC3 (Quze), PC6 (Neiguan), LR3 (Taichong), LR5 (Ligou)

3.4.2 SOP of manipulation

Traditional Chinese style acupuncture will be used and treatments are manipulated by specialized acupuncturists with at least five years’ training and three years’ experience.

The acupoints of all groups are asked to achieve *DeQi* sensation by lifting and thrusting combined with twirling and rotating the needles. All the groups are asked to retain the needles for 30 minutes, and close the acupoint holes with clean cotton balls to avoid bleeding when withdrawing the needle.

3.4.3 Treatment regimen

The total observation period within this study is 12 weeks for each patient. All patients should have recorded headache diaries for 4 weeks before randomization (baseline phase). After randomization the patients will receive 32 sessions of 30 minutes' duration over a period of 8 weeks (preferably 4 sessions in each week).

| -4 0 4（week） | | 8（week） |
| --- | --- | --- |
| baseline | treatment periods | |

### 3.5 fMRI scan

A standard eight-channel phase-array head coil is used, along with restraining foam pads to minimize head motion and to diminish scanner noise. Participants will be instructed to keep their eyes closed, relax, move as little as possible, and stay awake.

Scanning [parameters](app:ds:parameters): echo-planar imaging (EPI), TR=2000 ms, TE=30 ms, matrix=64×64, FOV=240 mm×240 mm, flip angle=90°, slice thickness=5 mm.

### 3.6 Outcome measurement

3.6.1 General information

gender, age, education level, vital signs, blood routine examination

These indicators will be filled and checked during the baseline.

3.6.2 Disease information

Patients met the inclusion criteria need to record the following information：

(1) disease history, family history, history of acupuncture treatment；

(2) headache diary：All patients fill in headache diaries during 4 weeks before randomization (baseline phase) and 4 and 8 weeks after randomization. The time of attack and stopping of headache, the intensity (VAS), the frequency, the location and the cause of headache, associated symptoms in each migraine attack. The patients document whether or not they take the medicine during treatment periods, and if patients take medicine they have to document the name, dosage of the medicine, the time of taking medicine, the time of relieving pain and the side effect of the medicine.

(3) others：the 6-item Headache Impact Test (HIT-6), the Zung Self-Rating Anxiety Scale (SAS), the Zung Self-Rating Depression Scale (SDS): document in the baseline and 4 and 8 weeks after randomization

(4) *Deqi* sensations: the feelings of *De-qi* will be collected after removing the needles during the 8^th^, 16^th^, 24^th^, and 32^nd^ sessions

### 3.7 Adverse events

Any adverse events, and how they are addressed, are recorded during the treatment periods. These adverse events include bleeding, hematoma, fainting, serious pain, and local infection. If patients suffer any serious adverse events, all details will be documented and reported to the IRB.

### 3.8 Statistical analysis

Clinical data analysis: Baseline data, demographic data and scale data will be analyzed by the SPSS 14.0 statistical software (SPSS Inc., Chicago, IL, USA). All analyses were based on the PP population. Chi-square test will be used to examine differences between groups (95% CI, 2-sided). The general linear model repeated measures procedure was used to test the differences in the repeated continuous variables (*Deqi* sensations) between the two groups.

Functional MRI image analysis: After data preprocessing conducted on SPM5 software, regional homogeneity and functional connectivity analysis will be performed to detect the modulation of different acupuncture interventions on pain-related functional networks at two time-points (prior to acupuncture, and after acupuncture treatment).
